# Evaluation of the Performance of Health Extension Workers in HIV-1/2 Screening Tests: A Comparative Cross-Sectional Study, Addis Ababa, Ethiopia

**DOI:** 10.1155/2020/7841352

**Published:** 2020-11-19

**Authors:** Mulatu Gashaw, Samuel Kindie, Minale Fekadie, Kassu Desta, Dawit Wolday

**Affiliations:** ^1^School of Medical Laboratory Science, Jimma University, Jimma, Ethiopia; ^2^Department of Medical Laboratory Science, Addis Ababa University, Addis Ababa, Ethiopia; ^3^School of Biomedical Sciences, Jimma University, Jimma, Ethiopia; ^4^Faculty of Medicine, Mekele University, Mek'ele, Ethiopia; ^5^Medical Biotech Laboratories, Addis Ababa, Ethiopia

## Abstract

**Background:**

Human resources for health-care delivery are essential for attaining global health and development goals. Especially in developing countries, health extension workers are frontline health personnel who can play a key role in preventing and controlling HIV/AIDS. This study aimed to evaluate the performance of health extension workers in HIV-1/2 screening tests. *Methodology*. A comparative cross-sectional study was carried out to evaluate the performance of health extension workers in HIV-1/2 screening tests. Study participants had performed HIV screening tests on the prepared sample panels. Finally, the percentage of accuracy, error rate, sensitivity, specificity, predictive values, and measure of agreement (kappa) were calculated using SPSS version 26.

**Result:**

Totally, 1600 HIV screening tests were performed, and of these, 684 and 235 tests were done by HEWs (*n* = 15) and laboratory personnel (*n* = 5), respectively, with three discordant results by HEWs from a single sample panel which was weak reactive for HIV antibody test. The sensitivity, specificity, PPV, and NPV of HIV screening tests by HEWs were 97.4%, 100%, 100%, and 97.22%, respectively, and 100% for all parameters when it is tested by laboratory professionals. The measure of kappa agreement was 0.971 (95% CI, 0.932–1) for HEWs and 1 for laboratory personnel compared with the reference result.

**Conclusion:**

Based on this evidence, we conclude that the potential contribution of HEWs can be invaluable in the expansion of HIV screening tests nationwide to compensate the shortage of laboratory personnel.

## 1. Background

Human resources for health-care delivery are essential for attaining global health and development goals [1, 2]. There is a strong correlation between the density of the health workforce, service coverage, and positive health outcomes, and a shortage of health workers has been widely and consistently identified as a barrier to the delivery of health services [1–4]. The HIV pandemic has increased the demand of health workers, and at the same time, the increased global commitment to HIV puts more pressure on bottlenecks created by health workforce shortages [5].

Health extension is an approach of promoting change through demonstration, working with opinion leaders and community-based educational activities [6]. Health extension workers (HEWs) are primarily responsible to achieve this program by working with the families and community at a grass root level to promote health and prevent disease through awareness creation [7]. Therefore, HEWs are frontline health personnel who can play a key role in increasing HIV/AIDS diagnostic services in the community [8, 9].

Rapid HIV screening tests have been developed predominantly for quick, easy to use, reliable onsite antibody testing for HIV by nonlaboratory-trained health professionals. The introduction of HIV screening tests to resource-limited countries could resolve many logistical issues [10]. Currently, HIV screening tests are widely used in nonlaboratory settings by nonlaboratory-trained operators [11]. Human immunodeficiency virus (HIV) screening testing is a key tool to fight against HIV/AIDS pandemic in low-income countries. However, a quality assurance program is very essential to ensure the quality of HIV screening test service outcomes [12–14]. Different research studies showed that transport costs are often a barrier to access VCT. Therefore, HIV prevention and control programs that aim in helping more people should consider home-based VCT services, especially in resource-limited settings where transport to a clinic and confidentiality are considerable barriers to access the service [15–17].

In countries, severely affected by HIV/AIDS pandemic, shortage of health workers present a major obstacle to scaling up HIV services [1]. To cope with the existing needs, nonlaboratory personnel should be participated in performing HIV screening tests [18]. Therefore, this study aimed to evaluate the performance of HIV screening tests by health extension workers in comparison with laboratory personnel.

## 2. Methods

### 2.1. Study Design and Setting

A comparative cross-sectional study design was carried out to evaluate the performance of HIV-1/2 screening tests by health extension workers compared with laboratory personnel in Addis Ababa, Ethiopia.

### 2.2. Study Participants

From 1375 health extension workers in the city, 15 HEWs from two subcities and five laboratory personnel from Addis Ababa University were taken conveniently. A total of twenty participants (HEWs = 15 and laboratory personnel = 5) were enrolled in study, and every one of them were performed HIV screening tests on 40 test panels based on the national HIV test algorithm of Ethiopia, and a total of 919 tests were done by both groups.

### 2.3. Panel Preparation and Dispatching

Ninety-two leftover sera samples were collected from St. Paul Hospital found in Addis Ababa and tested using rapid HIV test kits. Once the result was known, the sera were pooled, and a totally of forty sample panels were prepared. All the prepared sample panels were confirmed by fourth-generation ELISA, Murex HIV Ag/Ab Combination (Bio Murex, UK), for their HIV result again before used for the study. Finally, thirty-five negative and five positive sample panels were prepared to be tested by study participants and stored at 2–8°C till being tested by the participants.

### 2.4. Data Collection

After short demonstration about rapid HIV testing was given to HEWs, they have examined all 40 panels for the presence or absence of HIV-1/2 antibodies using three rapid HIV test kits according to the national HIV test algorithm, and finally they have recorded and reported their result to the investigators.

### 2.5. Data Analysis

Reported data were compared with the reference result and categorized as correct or incorrect and entered in Microsoft Excel sheet and transferred to SPSS version 26 for analysis. The kappa measure of agreement test, sensitivity, specificity, positive predictive value, negative predictive value, percentages of correct test results, and error rates were analyzed using SPSS software. Based on the calculated results, error rate <5% between each rapid test kit was considered as tolerable.

### 2.6. Ethical Consideration

Ethical approval was obtained from Department Research and Ethical Review Committee (DRERC) of Medical Laboratory Science, and the participants were asked to fill the written informed consent prior to enrolling in the study.

## 3. Results

### 3.1. Sociodemographic Characteristics of Participants

A total of 20 participants were enrolled in the study, and all of them have responded as they were very confident and comfortable to perform HIV screening tests. They also responded that their preference of training was demonstration in person. All health extension workers have taken at least one HIV-related training (Table 1), and their performance in HIV screening tests was correct except three of the HEWs. Of those HEWs, who had incorrect HIV screening tests, one has taken all the listed trainings related to HIV, and the other two took counseling and VCT (Table 1).

### 3.2. Performance of HEWs on Rapid HIV Tests against Laboratory Personnel

All HIV screening tests done by health extension workers, and skilled laboratory personnel were evaluated against the reference result which was confirmed by fourth-generation ELISA, Murex HIV Ag/Ab Combination (Bio Murex, UK). The performance of laboratory personnel was 100% for all parameters, while the performance of health extension workers was calculated, and the sensitivity, specificity, positive predictive value, and negative predictive were 97.4% (95% CI, 97–100%), 100%, 100%, and 97.2%, respectively, for all sample panels (Table 2). The kappa agreement of HEWs with laboratory personnel and reference result is 0.97(95% CI, 0.9–1) which shows a perfect agreement (Table 2).

**Figure 1:**
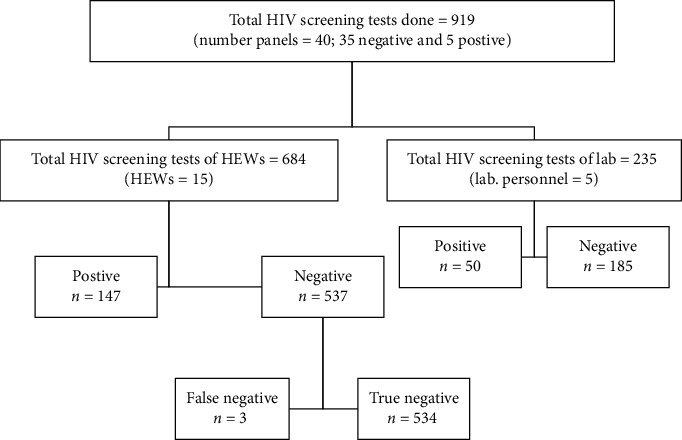
Prototypical STARD diagram to report HIV screening test results of health extension workers and laboratory personnel on pooled panels.

Totally, nine hundred and nineteen (919) rapid HIV tests were performed and reported with 916 (99.7%) concordance and 3 (0.3%) discordance results from a single sample panel by three of the health extension workers comparing with reference results using three rapid HIV test kits. The panel which was misdiagnosed by health extension workers was positive (reactive by any of the test kits) with faint red line, but both lines are not the same intensity that is normally taken as weak reactive. Except for those three health extension workers who scored a single error results each, all the others (*n* = 12) scored concordant results with laboratory personnel and reference results (Figure 1).

## 4. Discussion

According to WHO, the minimum required specificity and sensitivity for rapid HIV screening tests are at least 98%, and 99%, respectively [13, 19]. The current study showed that the overall performance of health extension workers on these rapid HIV screening tests is comparable with WHO recommendations. On the other hand, the sensitivity and specificity of HEWs on rapid HIV screening tests were greater than the study done in other African countries (sensitivity = 92.5% and specificity = 97.5%) [20]. These differences in HIV screening test performance between health extension workers and laboratory personnel were probably due to lack of experience for weakly reactive samples.

Even though different research studies showed that rapid HIV screening tests could be performed and read with accuracy by nonlaboratory personnel [12, 21, 22], our finding showed that all three errors were reported by HEWs from a single sample panel with faint red line which is commonly called as weak reactive. This finding is agreed with a study done in Congo which showed that, with a proper training, nurse counselors' produced false-negative results in weakly reactive samples [23].

An other study done in South Africa showed that the sensitivity and specificity of rapid HIV screening tests performed by nurses/counselors were 92.5–97.3% and 97.6–98.2%, respectively, but 100% for all parameters when performed by laboratory technicians [24], which was comparable with our finding. The discrepancy in test performance between nonlaboratory personnel and laboratory personnel was probably due to a lack of experience in weak reactive samples.

In our finding, the concordance and discordance test results of HEWs are 99.6% and 0.4%, respectively which is in agreement with studies done in African countries by Plate et al. who reported that the concordance between onsite rapid HIV screening test results, and results by laboratory personnel ranged from 95.7–99.5% (median: 98.7%) [20]. The discordance rate of our finding (0.4%) was less than the research studies done in other African countries and United States of America whose discordance rate was ranged from 0.4–1.1% and 2.1–4.6%, respectively [20, 25]. The error rate (0.4%) of our finding is also less than WHO recommendations, which says that it should not exceed 5% [13]. Therefore, the performance of HEWs in our study is acceptable to perform rapid HIV screening tests.

## 5. Conclusion

Based on the evidence from the current study, we conclude that where there is the necessary support, the potential contribution of HEWs can be optimized and represents a valuable addition to the urgent expansion of human resources for health, specifically for HIV services, nationwide.

## Figures and Tables

**Table 1:** Proportions of health extension workers who have taken HIV-related training before and percentage of their performance.

HIV-related training	No. of participants (%)	Percentage of concordant results no. (%)	Error rate (%)
Participants took all training	4 (26.7)	179 (99.4)	0.6
Participants took all except testing	1 (6.7)	45 (100)	0
Counseling, VCT and PMTCT	4 (26.7)	190 (100)	0
Counseling and VCT	3 (20.0)	133 (98.5)	1.5
Counseling	1 (6.7)	47 (100)	0
VCT	2 (13.3)	90 (100)	0
Total	15 (100)	684 (99.6)	0.4

**Table 2:** Comparing group performance of health extension workers with laboratory professionals on HIV rapid tests ^*∗*^.

Groups	Sensitivity (%)	Specificity (%)	PPV (%)	NPV (%)	Kappa agreement
HEWs	97.4 (97–100)	100	100	97.2	0.97 (0.9–1)
Lab. professionals	100	100	100	100	1

^*∗*^ Against gold standard as assessed using ELISA, *a*. All laboratory personnel and twelve HEWs who did not have error results, *b*. Three HEWs who have made one error result in each.

## Data Availability

The data used to support the findings of this study are included in the manuscript.
